# Empagliflozin Induces Transient Diuresis Without Changing Long-Term Overall Fluid Balance in Japanese Patients With Type 2 Diabetes

**DOI:** 10.1007/s13300-018-0385-5

**Published:** 2018-02-27

**Authors:** Atsutaka Yasui, Ganghyuck Lee, Tetsuaki Hirase, Tatsuroh Kaneko, Stefan Kaspers, Maximilian von Eynatten, Tomoo Okamura

**Affiliations:** 1 Nippon Boehringer Ingelheim Co. Ltd., Tokyo, Japan; 2 Eli Lilly Japan K.K., Kobe, Japan; 3 Boehringer Ingelheim Pharma GmbH & Co. KG, Ingelheim/Rhein, Germany

**Keywords:** Dehydration, Diuresis, Empagliflozin, Japanese patients, SGLT2 inhibitors, Type 2 diabetes, Urine volume

## Abstract

**Introduction:**

Empagliflozin, a sodium glucose co-transporter 2 (SGLT2) inhibitor, ameliorates hyperglycemia in patients with type 2 diabetes (T2D) by inducing sustained glucosuria. Empagliflozin treatment was previously associated with a transient increase in 24-h urine volume in Caucasian patients with T2D, however comparable evidence in Japanese T2D individuals is scarce. We therefore assessed acute and chronic changes in 24-h urine volume and fluid intake with empagliflozin in Japanese patients with T2D.

**Methods:**

In this randomized, double-blind, placebo-controlled, parallel-group, multiple-dose, 4-week trial, 100 Japanese patients with T2D were randomized to receive either 1, 5, 10, or 25 mg empagliflozin or placebo once-daily. Changes from baseline in 24-h urine volume and fluid intake were assessed at days 1, 27, and 28 after the initiation of empagliflozin.

**Results:**

The 24-h urine volume and fluid intake were comparable across all treatment groups at baseline. Patients treated with either 10 or 25 mg empagliflozin (i.e., the licensed doses in Japan) showed a significant increase in 24-h urine volume compared to placebo at day 1 (mean change from baseline: + 0.83, + 1.08, and + 0.29 L/day in the empagliflozin 10 and 25 mg groups and the placebo group, respectively; both *p* < 0.001 vs. placebo). However, 24-h urine volume levels in the empagliflozin groups were comparable to placebo at day 27 and 28 (differences vs placebo < 0.1 L/day; *p* > 0.05). The 24-h fluid intake was comparable across all study groups throughout the entire study period. No events consistent with dehydration were reported during empagliflozin treatment.

**Conclusion:**

Treatment initiation with empagliflozin in Japanese patients with T2D was associated with transient diuresis; however, overall urine volume returned towards baseline levels within 4 weeks of treatment. These findings are consistent with a physiological, adaptive mechanism of the kidney to maintain overall body fluid balance in response to treatment initiation with a SGLT2 inhibitor.

**Trial Registration Number:**

NCT00885118.

**Funding:**

Nippon Boehringer Ingelheim Co., Ltd.

## Introduction

Sodium glucose co-transporter 2 (SGLT2) is abundantly expressed in proximal tubular cells and mediates approximately 90% of glucose reabsorption within the kidney. SGLT2 inhibitors are a new class of oral antidiabetic drugs (OADs) that specifically target glucose reabsorption in the kidney, thereby facilitating glucose excretion into urine. This removal of excess glucose from the body is associated with a reduction in blood glucose levels in patients with type 2 diabetes (T2D) [1]. Moreover, the inhibition of glucose reabsorption mediated by SGLT2 inhibitors leads to increased osmolarity within the tubular lumen. Consequently, previous clinical studies with SGLT2 inhibitors have reported that treatment initiation with this drug class induced diuresis and led to a transient increase in overall urine volume [2, 3].

Empagliflozin (Jardiance^®^; Boehringer Ingelheim), a highly selective SGLT2 inhibitor [1], is approved in Europe, Japan, and the US for the treatment of hyperglycemia in patients with T2D [4, 5]. Moreover, empagliflozin is the first OAD to demonstrate a significant reduction (of 38%) in the risk of cardiovascular (CV) death as compared to standard-of-care therapy in a large cardiovascular outcomes trial in patients with T2D and established CV disease [6]. In previous phase 2–3 clinical trials, treatment with once-daily empagliflozin significantly ameliorated hyperglycemia, irrespective of commonly used glucose-lowering background therapy, and was well tolerated overall in T2D patients, including those of Japanese ethnicity [7–10].

In a pharmacodynamic study of Caucasian patients with T2D, initiation of empagliflozin treatment led to a transient increase in 24-h urine volume [2]. However, respective data for Japanese T2D patients and further effects of empagliflozin on daily fluid intake were scarce. Therefore, in the post hoc analysis reported in the present paper, we assessed changes in 24-h urine volume, fluid intake, and fluid balance in Japanese patients with T2D after the initiation of empagliflozin treatment in a previously completed local, randomized, controlled phase 2 trial [8].

## Methods

### Study Design

**Fig. 1:**
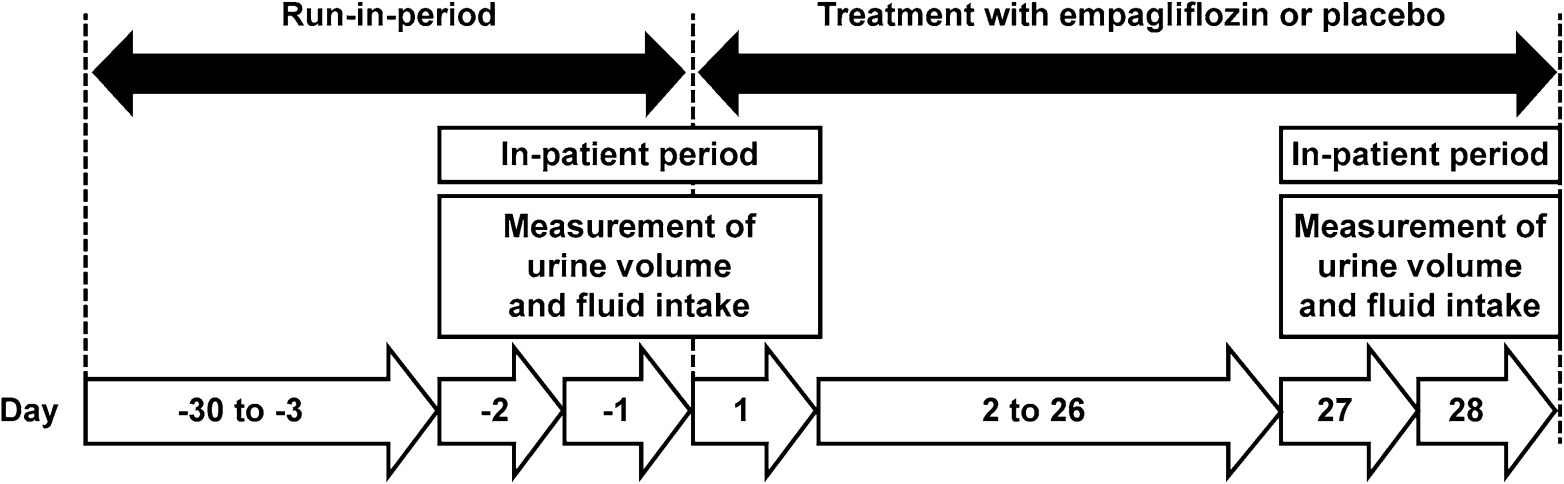
Study design depicting the run-in period and the on-treatment period

The trial design, eligibility criteria, and treatment allocations have been described previously [8]. Briefly, in this prospective, randomized, double-blind, multiple-dose, parallel-group, placebo-controlled clinical trial, 100 Japanese patients with T2D were randomized (1:1:1:1:1) to receive 1 mg (*n* = 19), 5 mg (*n* = 21), 10 mg (*n* = 20), or 25 mg (*n* = 19) empagliflozin or placebo (*n* = 21) once-daily for 4 weeks (NCT00885118). At baseline, all study participants were treatment naïve, allowing for a 30 days washout of previous glucose-lowering medications during the run-in period (Fig. 1). According to the study protocol, individuals were not eligible for this study if they had received concomitant diuretic background therapy. This report focused on the 10 mg and 25 mg empagliflozin treatment arms of the study, since they represent the approved doses of empagliflozin in Japan. The clinical trial (NCT00885118) was conducted in accordance with the Declaration of Helsinki (1996), the International Conference on Harmonisation Good Clinical Practice guidelines, and Japanese GCP regulations. Additionally, the local ethics committee (institutional review board) approved the study protocol. Written consent was received from all participants before study commencement.

### Assessments

Urine volume, fluid intake, and fluid balance were measured over 24 h on days − 2 (baseline), 1, 27, and 28 during the in-patient period (Fig. 1). Participants were instructed how to collect 24-h urine samples, and it was requested that containers with urine should be stored in refrigerators during all collection periods. The total weight of 24-h urine volume was calculated by subtracting the standardized weight of the empty container from the total weight of the container at the end of each collection period. Without further adjusting for urine gravity, 1 kg urine was assumed to equal 1 L. Study participants were allowed to consume fluids only after receiving approval from the investigators during the in-patient period, which facilitated the monitoring of fluid intake. Consequently, the daily fluid intake of the study participants was documented by investigators from 8:00 a.m. to 7:59 a.m. on the following day. The 24-h fluid balance was calculated as the difference between the 24-h urine volume and the 24-h fluid intake.

### Statistical Analyses

Descriptive statistics were used for all the endpoints. In this post hoc analysis, changes in 24-h urinary volume, fluid intake, fluid balance, and serum urea from baseline were assessed using an analysis of covariance model with treatment as a fixed effect and the baseline for each endpoint as a covariate. Treatment comparisons were done between the empagliflozin groups and the placebo group. Two-sided *p* values < 0.05 were considered significant.

## Results

### Patients

Among the total study population, 84 patients were male. Median age was 59.5 (range 34–70) years, and median body mass index (BMI) was 24.3 (range 18.0–39.1) kg/m^2^. Baseline 24-h urine volume and fluid intake, alongside other relevant clinical characteristics, were comparable across treatment groups (Table 1) [8].

**Table 1:** Baseline 24-h urine volume, daily fluid intake and other relevant clinical characteristics

	Placebo (*n* = 21)	Empagliflozin	
		10 mg (*n* = 20)	25 mg (*n* = 19)
Urine volume (l/day)	2.50 ± 0.81	2.34 ± 0.98	2.26 ± 0.82
Fluid intake (l/day)	1.92 ± 0.75	2.08 ± 0.83	1.78 ± 0.75
Age (years)	57.2 ± 10.0	55.8 ± 8.0	60.8 ± 8.7
Male [*N* (%)]	16 (76.2)	17 (85.0)	15 (78.9)
BMI (kg/m^2^)	26.14 ± 3.71	24.65 ± 3.63	24.16 ± 3.61
HbA1c (%)	7.97 ± 0.69	8.24 ± 0.74	7.97 ± 0.77
eGFR (mL/min/1.73 m^2^)^a^	98.36 ± 12.90	100.13 ± 15.97	99.58 ± 13.12
ACEi/ARB background [*N* (%)]	5 (23.8)	1 (5.0)	2 (10.5)
Diabetes duration [*N* (%)]			
≤ 1 year	4 (19.0)	1 (5.0)	2 (10.5)
≤ 5 years, > 1 year	7 (33.3)	9 (45.0)	5 (26.3)
> 5 years	10 (47.6)	10 (50.0)	12 (63.2)

All values presented as mean ± SD or number of patients (%)

*SD* standard deviation, *ACEi* angiotensin converting enzyme inhibitors, *ARB* angiotensin receptor blockers, *BMI* body mass index, *eGFR* estimated glomerular filtration rate, *HbA1c* hemoglobin A1c, *MDRD* modification of diet in renal disease

^a^The modified abbreviated MDRD formulae

### Urine Volume

**Fig. 2a–d:**
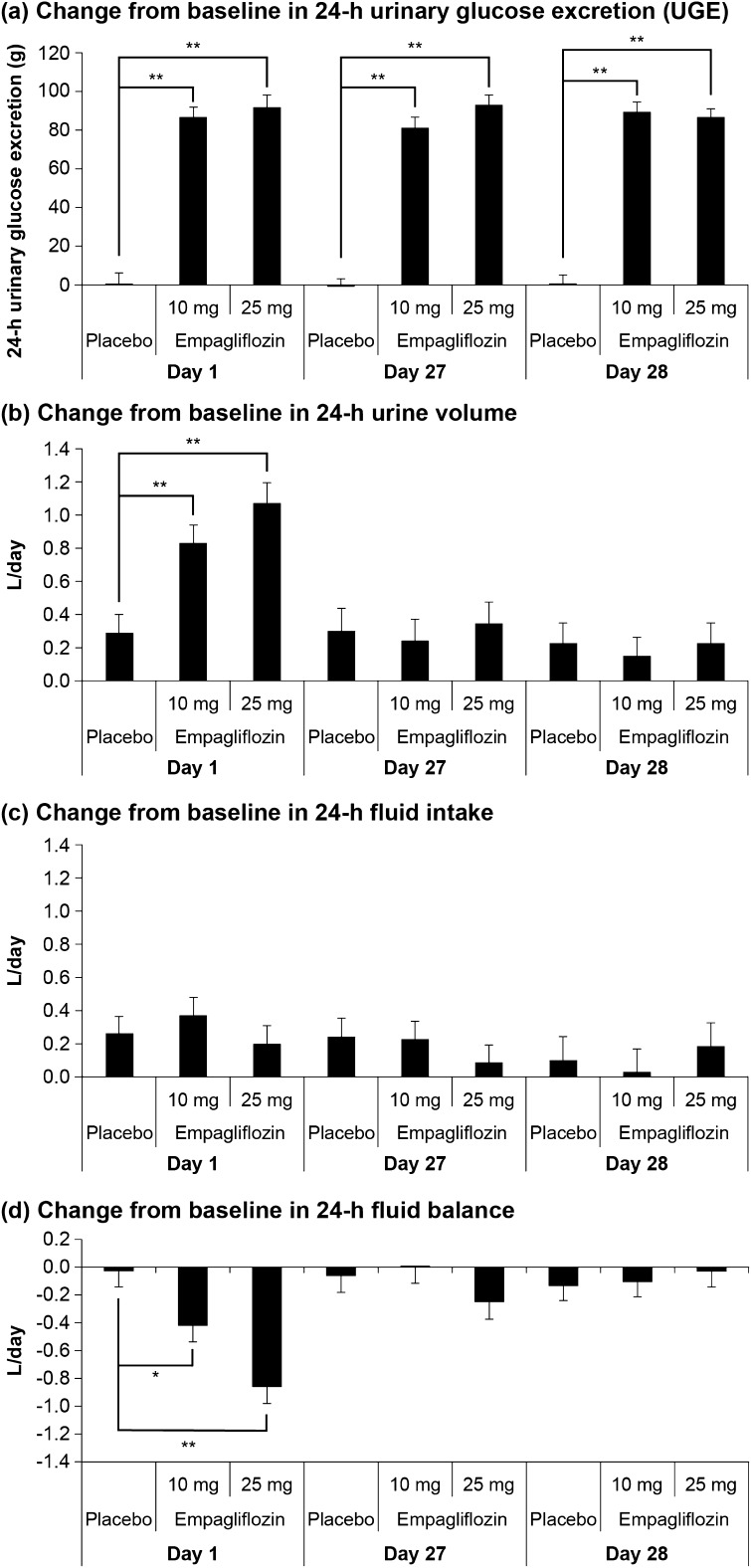
Effects of 10 mg and 25 mg empagliflozin on **a** the change from baseline in 24-h urinary glucose excretion (UGE), **b** the change from baseline in 24-h urine volume, **c** the change from baseline in 24-h fluid intake, and **d** the change from baseline in 24-h fluid balance. Data are shown as the adjusted mean + standard error (*n* = 18–21 per group). *p < 0.05, **p < 0.001. *L/day*: liters/day. Modified with permission from [8]

We previously reported that 24-h urinary glucose excretion (UGE) was significantly increased on day 1 with either empagliflozin 10 or 25 mg, and a significant increase in 24-h UGE was maintained throughout the active treatment period until day 28 (Fig. 2a) [8]. On day 1, 24-h urine volume increased significantly in patients treated with the 10 mg and 25 mg doses of empagliflozin compared to placebo (adjusted mean change from baseline at day 1 was + 0.83, + 1.08, and + 0.29 L/day in the 10 mg and 25 mg empagliflozin groups and the placebo group, respectively; both *p* < 0.001 versus placebo) (Fig. 2b). However, after the chronic treatment period of 4 weeks, the 24-h urine volume was comparable between the empagliflozin 10 mg and 25 mg groups and placebo on days 27 and 28, respectively (Fig. 2b).

### Fluid Intake, Whole-Body Fluid Balance, and Reports of Dehydration

The 24-h fluid intake was similar across the 10 mg and 25 mg empagliflozin groups and the placebo group on days 1, 27, and 28 (Fig. 2c). Whole-body fluid balance was altered in patients treated with empagliflozin 10 mg and 25 mg on day 1 (adjusted mean change from baseline at day 1 was − 0.42 and − 0.86 L/day in the 10 mg and 25 mg empagliflozin groups, respectively) but was unchanged in patients treated with placebo (Fig. 2d). After chronic treatment, at week 4, whole-body fluid balance was similar for both the empagliflozin groups and the placebo group on days 27 and 28 (differences vs placebo < 0.19 L/day; *p* > 0.05). No adverse events consistent with dehydration were reported for the empagliflozin 10 mg, empagliflozin 25 mg, and placebo groups during the entire on-treatment study period.

## Discussion

Since SGLT2 inhibitors induce sustained glucosuria, it has been speculated that increased osmolarity due to augmented glucose delivery to the tubular lumen of the kidneys could impact short-term and long-term whole-body fluid balance. Consequently, reports of adverse events consistent with volume depletion have emerged from clinical trials as well as the routine clinical use of SGLT2 inhibitors. We therefore assessed the acute and chronic effects of empagliflozin on 24-h urine volume production and daily fluid intake in Japanese patients with T2D using data retrieved from a previously completed phase 2, pharmacodynamic study [8].

Our results show that treatment initiation with empagliflozin leads to a rapid pharmacodynamic response which is reflected in a significant increase in UGE following the first intake of the study drug. This effect of empagliflozin was maintained throughout the study period, leading to stable and clinically relevant levels of glucosuria. Novel insights from this study show that patients initiated on either 10 mg or 25 mg empagliflozin presented a significant increase in 24-h urine volume on day 1. The observed increase in urine volume within the first 24 h of treatment corresponded to an average additional daily output of approximately 500–800 mL for the 10 mg and 25 mg doses of empagliflozin compared to placebo. Importantly, however, this increase in urine volume with empagliflozin was transient in nature, and returned to 24-h baseline urine levels similar to those observed in the placebo group after 4 weeks of chronic treatment. These results in Japanese patients with T2D are reminiscent of a previous study in Caucasian T2D patients treated with empagliflozin [2]. Data showed a significant increase in 24-h urine volume with empagliflozin on day 1, but urine volume returned to baseline values, with no change versus placebo as early as day 5 despite sustained glucosuria. Similar data were also reported with another SGLT2 inhibitor, canagliflozin, with an initial increase in urine volume versus placebo observed in Japanese patients with T2D treated with 25–400 mg canagliflozin on day 1; this effect disappeared as early as day 2 [11].

Our data address an important clinical consideration pertaining to the appropriate use of SGLT2 inhibitors in patients with T2D. Since the mechanism of action of SGLT2 inhibitors is known to induce sustained glucosuria, concerns have been raised that patients could be at increased risk of a clinically relevant loss of plasma and/or whole-body fluids, leading to volume depletion or dehydration events. Such concerns are of clinical relevance to Japanese T2D patients due to important demographic differences such as a higher average age and a lower BMI as compared to Caucasian T2D populations. The existing evidence for SGLT2 inhibitors to date, including studies in Japanese T2D patients, indicates that an increase in urine volume occurs immediately after treatment initiation (i.e., during the first 24–48 h). Hence, patients may be advised to increase fluid intake accordingly and to monitor acute changes in both urine output and body weight when starting a SGLT2 inhibitor. After this short-term initial phase of increased urine production, equilibrium is expected to be reached within the first week of SGLT2 treatment [2, 3, 11]. Notably, comprehensive studies of patients at increased risk for dehydration (e.g., T2D individuals > 75 years old and/or receiving concomitant diuretic therapy) are currently scarce, so careful weighing of the benefits and risks of SGLT2 inhibitors, including the clinical risk of volume changes in these patients, is warranted.

How could the transient effect of SGLT2 inhibitors on urine volume (despite the fact that glucosuria is sustained at levels of approximately 50–80 g/day [12], exerting osmotic forces along the nephron) be explained? It is tempting to speculate that there must be a holistic compensatory mechanism of the kidneys that is capable of mitigating the risk of excess whole-body fluid loss by osmotic diuresis. This hypothesis seems obvious given the central role of the kidneys in maintaining whole-body fluid and electrolyte balance. Recently, however, experimental data have permitted novel insights into the more specific nature of SGLT2 inhibitor-related adaptive responses within the kidney. In a study of Sprague–Dawley rats with streptozotocin-induced diabetes, dapagliflozin induced the expression of urea transporter A1 (UT-A1) in the inner medulla of the kidney, resulting in decreased urea excretion in the urine [13]. The authors speculate that SGLT2 inhibition may in turn increase urea levels in the medullary interstitium, which would augment outbound osmotic forces that are capable of reabsorbing free water from the tubular lumen [13]. In accordance with this hypothesis, we observed a mild increase in serum urea level with empagliflozin in our study [adjusted mean difference (95% confidence interval (CI)) versus placebo at day 28: empagliflozin 10 mg, 0.383 (0.259, 0.506) mmol/L, *p* < 0.0001; empagliflozin 25 mg, 0.375 (0.248, 0.502) mmol/L, *p* < 0.0001]. These results are in accordance with evidence from a previous Caucasian study with empagliflozin [2]. Therefore, the combined experimental and clinical data suggest that SGLT2 inhibitor-mediated osmotic diuresis could be compensated, at least in part, by an increase in the urea level and hypertonicity in the inner medulla, enabling the kidney to maintain whole-body fluid balance even under conditions of sustained glucosuria.

Although vasopressin plays a pivotal role in the regulation of urine volume [14], a previous study by Tanaka et al. showed that treatment with canagliflozin had no effect on vasopressin levels in patients [3], suggesting that the alterations in urine volume mediated by SGLT2 inhibitors could be attributed to vasopressin-independent mechanisms. Future research is needed to further decipher the nature of potential additional tubular compensatory mechanisms, including the expression and activity of transporters for solutes, electrolytes, and free water.

The strength of our study is based on its randomized, double-blind, placebo-controlled design and the consistency of findings for the 10 mg and 25 mg empagliflozin groups. Assessments of 24-h urine volume and fluid intake occurred during in-patient periods, increasing the internal validity of our findings. Limitations apply to the study population, which did not include patients at a particularly increased risk for dehydration (e.g., higher age and concomitant diuretic use). In addition, 24-h urine collections were performed on day 1 and then again after 4 weeks of treatment. Hence, it is still expected to obtain a more detailed time course for the observed adaptations in daily urine volume, especially during the first 1–2 weeks of treatment with empagliflozin in Japanese patients with type 2 diabetes.

## Conclusion

Empagliflozin was associated with a transient increase in diuresis in Japanese patients with T2D within the first 24 hours of treatment. Thereafter, urine volume returned towards placebo levels after 4 weeks of therapy with empagliflozin. Neither an increased daily fluid intake nor events consistent with dehydration were observed with empagliflozin throughout the 4-week treatment period. Our findings support an early and effective compensatory mechanism of the kidney that  in order to maintain whole-body fluid balance, despite sustained glucosuria, in Japanese T2D patients treated with empagliflozin. Clinical evidence relating to empagliflozin supports safe and effective treatment initiation in appropriate Japanese patients with T2D without an excess risk of undesirable volume-related events.
